# Mr. Ring, on Bronchocele

**Published:** 1801-01

**Authors:** John Ring

**Affiliations:** New Street, Hanover Square


### *To the Editors of the Medical and Phyfical Journal.*

Gentlemen,

I Lately tranfmitted fome obfervations on Bronchocele, which you have done me the honour to infert in the laft Number of your very refpectable Journal. I beg leave to correct an error in the formula recommended for the complaint. Inftead of directing the mafs, containing two ounces of burnt fponge, to be divided into forty-eight, as I ought to have done, I directed it to be divided into twenty-four. This inadvertence probably arofe from my knowing the perfectly harmlefs nature of the remedy; and muft be apparent to every reader, from my ftating in another part of my paper, that every lozenge is to contain a fcruple of the medicine.

This is the dofe I have hitherto given, and, except in one inftance, only twice a day. I intend to increafe the quantity to half a drachm, or more, in cafe the patient can bear it. The only difficulty arifing from it is, that from the flavour, and its being held in the mouth till it diffolves, naufea is excited in fome delicate habits; fuch as thofe commonly are who are liable to this difeafe.

I am more and more convinced that the treatment of this diforder is not well underftood, having been informed by different medical men, fince the publication of my former paper on this fubject, that in the courfe of their obfervation and practice, they had known but little benefit derived from the means employed for its cure.

From a gentleman of great eminence in the profeffion I learn, that his friend, the late Dr. Bromfield, died of this complaint; and that the tumour was opened about a week previous to his death, an operation which ferved only to prolong a miferable exiftence.

In perfons rather advanced in years, the Bronchocele will not always yield to medicine; but its commencement frequently takes place early in life; and, if due attention is paid to it in time, I have reafon to believe that it will generally prove curable. Particular care fhould be taken that the medicine is not left off too foon; for the diforder is of an infidious nature, and apt to deceive thofe who are not much accuftomed to it. I have feveral times known patients leave off their medicine in this cafe, from an idea that the Bronchocele was cured; and, after a while, they have been obliged to have recourfe to it again. It is, however, poffible that the difpofition only to the difeafe might remain, and caufe the tumour to regenerate. Be this as it may, the effect of returning to the ufe of the lozenges has been the fame; if properly perfevered in they have feldom failed to exterminate the complaint.

To thefe remarks I beg leave to fubjoin a caution to thofe perfons who venture to give quack medicines. A phyfician lately mentioned a cafe at a medical fociety, in the prefence of one, at leaft, of the Editors of this Journal, which ought to ferve as a warning to all ranks of perfons in this age of empiricifm.

This cafe I fhould not have prefumed to communicate to the world, had I reafon to believe that gentleman himfelf would publifh it; but he gave the fociety reafon to conclude he had no fuch intenfion, by faying, that he was not much in the habit of publifhing. This is to be regretted in a practitioner of eminence, and efpecially in one who has fingular opportunities of feeing uncommon cafes.

Whether this averfion to appear often in print arifes from diffidence, or want of leifure, I pretend not to determine; but, as there are fo many refpectable channels open for the ready communication of ufeful cafes, I fincerely wifh it was in my power to prevail on other diftinguifhed members of the profeffion to follow the example of a Denman; and not confider it as any difparagement of their dignity to publifh a fingle cafe, or a fingle ufeful remark, in any vehicle of information which may be likely to fall into the hands of medical practitioners at large, and to prove beneficial to the world.

The cafe to which I now allude was one in which Dalby's Carminative, given in too large a dofe, proved fatal. The dofe, according to the nurfe's account, was a tea fpoonful; the age of the child about a twelvemonth. Upon examination after death, the injury appeared to have been done to the brain; a remarkable degree of congeftion appeared in every finus, and every veffel of the pia mater, fo as to leave no room to doubt that the caufe of death was feated there.

This, and other cafes, where exceffive dofes of opium have proved deftructive, as well as one publifhed in your Journal by Mr. Brookes, where a fatal confequence was prevented by venæfection, feem to confirm the propriety of that gentleman's practice; practice; and to ſhew that unloading the veſſels of the head is one of the chief indications of cure.

I have known Storey's worm cakes prove fatal; and, what made the caſe ſtill more affecting, they were given by a father to a child, who had never ſhewn a ſingle ſymptom of having worms.

In regard to Dalby's Carminative it is neceſſary to remark, that the phyſician who mentioned the fatal caſe, alſo related an inſtance in which it proved very pernicious to an adult; who, having been uſed to take it for a complaint of the ſtomach or bowels, ſent for a bottle to a different ſhop from that where ſhe uſed to buy it, and it proved ſo much ſtronger than what ſhe had been accuſtomed to take, that, with the able aſſiſtance of this experienced practitioner, ſhe did not recover without much difficulty. I am,

GENTLEMEN,

Your moſt humble ſervant,

JOHN RING.

*New Street, Hanover Square,*

